# Cork Composites: A Review

**DOI:** 10.3390/ma2030776

**Published:** 2009-07-16

**Authors:** Luís Gil

**Affiliations:** Instituto Nacional de Engenharia, Tecnologia e Inovação, I.P., Unidade de Tecnologia da Cortiça, Estrada do Paço do Lumiar, 1649-038 Lisboa, Portugal; E-Mail: luis.gil@ineti.pt; Tel. +351210924757; Fax: + 351217166939

**Keywords:** cork, composites, cork agglomerates, cork applications

## Abstract

Cork is a material which has been used for mankind for the last 5,000 years and it is a strategic material used for multiple applications, from wine bottles to aeronautics. Many of current cork materials are composites, in particular cork materials for floor and wall coverings and several other building and industrial applications. Recent developments in cork research have shifted from the classical cork-wine relationship to quality and environmental issues, exploitation of cork industry residues and new cork based materials. In recent years a number of new cork based composite materials were developed.

## 1. Introduction

**Figure 1:**
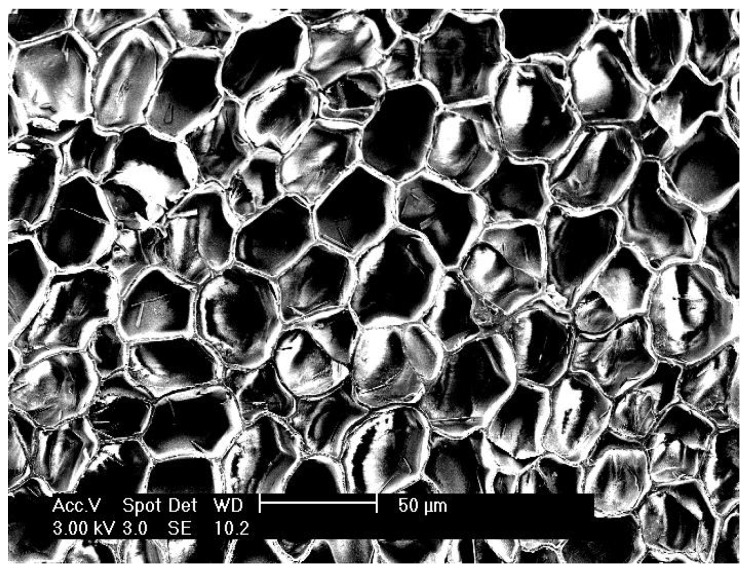
Cork cellular structure.

Cork is the suberous covering (suberose parenchyma, or bark) of the species *Quercus Suber* L., commonly known as the cork oak. It is composed of an aggregate of cells, about 42 million per cubic centimeter, which have five wall layers. Cork is one of the most versatile natural raw materials known. Cork is a very lightweight material, elastic and flexible and impermeable to gases or liquids, imperishable and good electric insulator, as well as thermal, sound and vibration insulator [1] and a dielectric material. As a cellular material its unique properties arise from its closed cell structure (see Figure 1).

The European Union, and particularly the southern Mediterranean countries, is the World’s major producer of cork. Portugal, which holds about 60% of the total cork tree area provides about 80% of the cork produced in the World. Cork forests are extremely well adapted to Europe’s southern semi-arid regions, preventing desertification and being the perfect habitat for many animal and plant species.

Cork’s low thermal conductivity combined with a reasonable compressive strength makes it an excellent material for thermal insulation purposes and when compressive loads are present. Its friction (anti-sliding) properties make it also good for floor coverings or in handles. Today cork products are used for thermal insulation in refrigerators and rockets, acoustic insulation in submarines and recording studios, seals and joints in woodwind instruments and combustion engines, and as energy-absorbing medium in flooring, shoes and packaging, and of course as stoppers [1].

As cork stopper production (natural corks) is only able to use until at most 25% of the raw material, new applications were sought. Cork composites are part of the current cork derivatives and are one of the most promising fields of cork technology evolution.

## 2. Historic Survey

Since the early stages of development of cork processing to obtain natural cork stoppers, it was noted that a huge volume of cork waste material was produced and there was a need of its utilization [2]. Composite materials including cork were a way to fulfill this need.

At the end of the XIX century, an American manufacturer of lifejackets accidentally found that it was possible to produce self-agglomerated cork (nowadays also called insulation corkboard or ICB). This was the beginning of a new world of opportunities in which waste cork and cork previously thought to be of no commercial value could also be used [1].

This period was also responsible for another invention. In the United Kingdom, Frederick Walton invented linoleum for floor covering. This was discovered by chance when he mixed oxidized linseed oil with very fine milled cork waste and pressed this mixture. At this time another material for floor covering was already been produced in UK, named “kamptulicon” and made of ground rubber and cork mixed with gums and pressed. The invention of composition cork in 1909 is due to Charles McManus, who used natural glues to bind the cork granules. At this time references were also made to the use of tar and pitch. The first agglomerated cork stoppers were developed in the beginning of the XX century using several types of glues (dextrin, casein, gelatin, urea-formaldehyde, amine) and in 1968 polyurethane [2]. Several undocumented experiments were carried out at an industrial level and lead to many of the current commercial cork composites.

## 3. Current Cork Composites in the Market

In the market there are several types of cork agglomerates. Cork agglomerates are divided into two categories: composition cork and insulation corkboard. The second category is made of only cork without any external binding agents or any other added material and so it cannot be considered as a true composite material and will therefore not be discussed in this review. Composition cork is made by the binding of cork particles with different binders (polyurethane, melamine, rubber etc) yielding products such as agglomerated cork stoppers, floor coverings, joints, etc. The physical and chemical characteristics of the binders determine the strength of agglomerate and therefore its applications [3]. The production and characteristics of current cork composites will now be described.

Waste cork from the stopper industry, low quality cork (refuse) and finally virgin cork are all used to produce cork granulates. These are separated and classified according to density and grain size. The finest are used to produce linoleum. These cork granulates can be used as a final product in several applications or used as raw material for the production of composition cork [1]. Accordingly, composition cork is made of granules which have been joined together using different synthetic or natural binding agents (usually urethane, melaminic and phenolic resins). The granules with a specific granulometry and volumetric mass are placed into a mixing device (shovel or helicoidal mixers) for automatic or manual dosage. A mixture of cork granules and glue and/or other additives are put into a mould (usually metallic and paralelipipedic in shape or cylindrical for rolls) which is then closed and heated, usually at more than 120 ºC and in tunnels, for 4-22 hours, in order to produce a block which upon cooling (or not) is then sliced into sheets, which are then dimensionally finished. By using various binding agents and chemical additives it is possible to adapt the grade to suit user requirements and the purpose for which the material is to be used. For example, wall coverings have a density of 200-300 kg/m^3^ and floor coverings of 400-500 kg/m^3^ [1]. These products are usually produced in sheets, rolls, blocks, or tiles, with different thickness, densities and finishing: simply polished, waxed, painted, varnished or covered with a vinyl layer or even extruded or moulded. The group with a vinyl layer may use a decorative sheet between the PVC (polyvinyl chloride) and the agglomerate underneath. A cork layer associated to a MDF (medium density fiberboard) base, for example, constitutes a new type of flooring, known as floating floor coverings [1]. As mentioned before, the finest cork granules are used to produce linoleum, which contains linseed, resin, lead or magnesium oxide and coloring agents. Linoleum is resistant to wear and tear and easy to clean, like all the other cork coverings [1]. 

**Figure 2:**
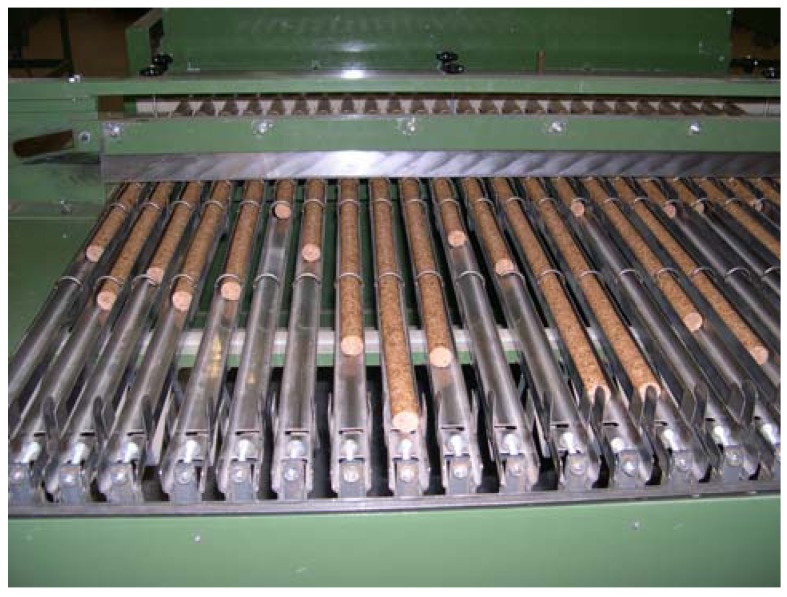
Agglomerated cork baton for agglomerated cork stopper production.

The granulation of cork for the production of agglomerated cork stoppers uses cork waste (boiled cork) from the cutting stage (up to 90% of the granulated material) or material rejected at the stopper sorting stage. Contaminated material must be avoided [1]. Agglomerated cork stoppers consist of small pieces of natural cork bound together into a single stopper in individual moulds or agglomerated cork batons which are then cut in individual stoppers (see Figure 2). There are simple agglomerated corks and two main types of composite stoppers, the stopper for champagne and sparkling wine (head in agglomerate and two or more disks in the bottom) and the “1+1” stopper for the other wines (body in agglomerate and one disk at each end) Simple agglomerate cork stoppers are usually edge finished and like the other agglomerated cork stoppers they may be cleaned and lubricated. Only FDA approved glues are used [1].

The production of cork rubber is similar to other rubber-like product production. Rubber and cork granules are mixed in rolls and the mats obtained are introduced into a mould, which is heated for polymerization. Usually blocks are obtained, but cylinders can also be obtained. The blocks are sliced and the cylinders are cut (unrolled) to produce rolls [1]. The heating process may take from several hours (in a typical oven) to some minutes (in microwave systems). Most common cork rubber materials use cork granulates of 60-70 kg/m^3^, in 15 to 260% by weight in relation to rubber. The main types of used rubbers are SBR (styrene-butadiene rubber), NBR (nitrile rubber), acrylic rubbers [1] and EVA (ethylene vinyl acetate). Cork rubber materials are mainly used in gaskets (combustion engines, etc.), vibration insulation and heavy duty coverings.

Most of other cork products are made by similar processes to the floor and wall covering cork products. For example, filler and expansion joints are specially designed to neutralize the expansion and contraction phenomena that can jeopardize concrete constructions, being an excellent protection against cracks, usually due to temperature variations. These materials can withstand continuous strain, whatever the moisture conditions are [1].

In buildings and other civil construction works, cork products may be used for thermal insulation, vibration insulation, acoustic correction, floor covering, wall covering, false ceilings, and expansion joints. Some of these cork products may be composed with other construction materials, for example, composition cork and MDF or HDF (high density fiberboard) and wood veneer. These floating floors are made by the attachment of the different layers by applying glue on both sides of the surfaces and the assemblage is carried out by plate pressing. Some specific applications of cork in the industrial environment are: cork slabs and sheets, pipe insulation, battery mold coating, cold storage insulation, machinery anti-vibration sheets, storage tank insulation etc. Cork rubber is resistant to wear, being non-slip and sound absorbent, resistant to oil grease and salts which makes it suitable for industrial and vehicle flooring. Cork makes good gaskets because it accommodates large elastic distortion and volume change and its closed cells are impervious to water and oils. The recovery capabilities of cork after compression are also important for gaskets, allowing a continuous pressure against both sealed surfaces. In footwear applications, cork materials are ideal to the technical demands of the shoe industry and can be used in insocks/insoles, heels, sole and bottom fillers, mid soles, coverings, footbeds (moulded applications). For example, in insocks cork materials enhance the comfort of the foot by giving excellent shock absorption, cushioning, ground insulation and impermeability. Besides the automotive and industrial applications, also the aeronautic and military industries are big consumers of cork derivatives, For example, there are protective heat shields in missiles and in the Space Shuttle made of cork. Fire retardant cork agglomerate is used in warships, and the internal lining of submarines [1,4]. Some multilayered materials were also developed, for example for flooring systems underlayments as e.g. in [5].

The production of cork agglomerates based on cork powder is difficult or even impossible due to its enormous superface area. So, to achieve an agglomeration technology to overcome these disadvantages would be very important.

Very few studies of the properties of natural cork and its derivatives concerning their electrical and dielectric properties are found in scientific literature. The electrical and dielectric properties of cork only recently have been studied. Measurements of isothermal charge and discharge currents from cork agglomerates are available [6,7]. The isothermal current characteristics and the conductivity of samples were investigated under different conditions [electric field, temperature and environmental conditions: in vacuum and in air at ambient relative humidity (RH)]. The samples could be conditioned (dried in P_2_O_5_ atmosphere at room temperature) or not. Cork is a good electrical insulator and because of the cells can be filled with gas it might be possible to use it as a porous dielectric, which can be electrically charged and is able to retain this charge. In this case it would behave as a piezoelectric and it could be used to develop smart sensors. The electrical properties of cork have been found to be related to the water content of the material. These properties were not just found in natural cork, but also in cork derivatives such as cork agglomerates and cork composites.

## 4. New Cork Composites

In this chapter new cork composites developed in the last years and which are not yet in the market are reviewed.

### 4.1. Sandwich cork composites

Cork based agglomerates are an ideal core material for sandwich components of lightweight structures, such as those used in aerospace applications [8]. Static bending tests and dynamic loads were carried out using carbon-cork sandwich specimens. The results from experimental tests revealed that cork agglomerates performance essentially depends on the cork granulate size, its density and the bonding procedure used for the cohesion of granulates, and these parameters can be adjusted. Optimized cork agglomerates have some specific properties which confirm their superior ability as a core material of sandwich components when compared with other conventional materials. The use of lightweight structures with high strength to weight ratio has been a constant feature in the transport industry, and the rising demand for new materials has resulted in a significant growth in the sandwich composite technology area. The properties of primary interest for the core materials can be summarised as: low density, high shear modulus, high shear strength, elevated stiffness perpendicular to the faces and both good thermal and acoustic insulation characteristics [9]. Some properties of cork agglomerates suggest that these materials can evince some remarkable properties when acting as a core of a sandwich component, namely a high damage tolerance to impact loads, good thermal and acoustic insulation capacities and excellent damping characteristics for the suppression of vibrations. A study was carried out in which at a first stage, several types of commercial cork agglomerates (with different granule sizes) were tested, showing poor mechanical performance when compared to conventional core materials. In order to improve the mechanical behaviour of cork as a core material, three new types of cork agglomerates were fabricated with conventional cork granulates but using epoxy resin as the adhesion element. Cork agglomerates developed with epoxy resin present significantly better core shear stress limits, even when compared with Rohacell^®^ rigid foam, which reduces the crack propagation region. This is an important achievement that can place cork-epoxy agglomerates at the leading edge of currently available materials used in sandwich structures. All produced cork based sandwiches presented considerably higher load values than those obtained for other type of high performance core materials (such as Rohacell^®^) and the extraordinary recovery capacity verified in the cork agglomerates sandwiches displacement curves is an exclusive and intrinsic characteristic of cork. Compared with high performance foams, sandwich components with enhanced cork agglomerates have a higher energy absorption capacity and, therefore, better crashworthiness properties when impact loading is expected. Cork agglomerates with lower densities present better thermal properties, which is an important issue when considering the design of mechanically efficient structures with low weight requirements (such as aerospace components) [8]. In this field other materials have been patented, based on layers of different materials in which one or several were made of composition cork, see e.g. [10,11,12].

### 4.2. Cork/beverage carton wastes composite

**Figure 3:**
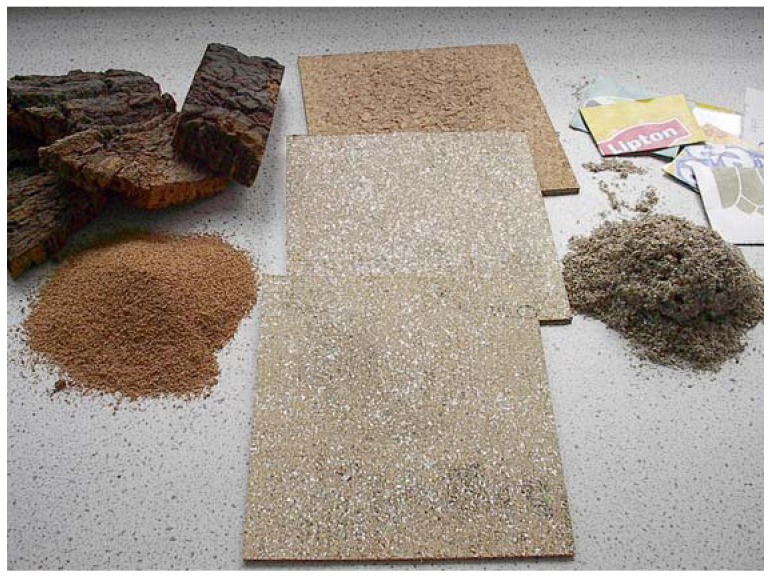
**C**ork, beverage cartons and composite samples.

A patented process for the production of composite agglomerates comprising a group of fibres and particles from the fragmentation and/or grinding of waste material, for example, waste material of packages composed by layers of plastic/card/aluminium sheets (beverage cartons) and particles of cork, without adding external binders, through pressing and heating during a time period sufficient to accomplish agglomeration and mechanical strength, was developed (see Figure 3). This process is preferably used with packaging wastes (for example beverage cartons, cork stoppers) but it can also be used with industrial wastes. It was noted that it was possible to produce interesting composite materials based on urban or industrial wastes without using additional binders, which have wide range of characteristics with interest for several applications. Other materials can also be included in the formulation and/or at the pressing operation one or both the surfaces of the board can be covered by a sheet of other material binding to the surface. The process allows compositions having wide ranges of *cork:beverage carton material* ratios (any proportion), giving rise to composites with very different characteristics for multiple applications (e.g. greater cork percentage for insulation applications and greater percentage of beverage cartons material for greater stiffness and mechanical resistance). Moulded pieces with different shapes can also be obtained. The new composites have a physical-mechanical behaviour which is similar to that of other materials suitable for a wide range of applications and these allow one to foresee utilizations as floor coverings, dividing panels, furniture and other similar applications. New studies on these composites are also foreseen, namely for applications as anti-electrostatic materials (e.g. floor coverings for computer rooms) due to the presence of an electrical conductor (aluminium) and also applications as smart materials. In particularly the piezoelectric characteristics that could lead to possible applications as piezoelectric sensors/actuators. Measurements of isothermal charge and discharge currents from cork/beverage cartons composite were made. The isothermal current characteristics and the samples conductivity were investigated under different electric field, temperature and environmental conditions (vacuum and in air at ambient relative humidity). The new composite mechanical and acoustical properties were also studied in order to compare with other available commercial materials also based in cork composites. These materials were also charged in order to investigate piezoelectric characteristics that could lead to the ability to store electrical charge. The main problem found was related to the water content in cork, only of a few percent in weight, but large enough to influence greatly the conductivity of cork and, consequently, the charge storage capability. To overcome this problem cork has been combined with hydrophobic materials. In this work a commercial wax (paraffin wax) was used to produce a cork/paraffin composite by hot pressing. After milled and mixed natural cork, TetraPak® containers waste and paraffin were pressed to make plaques of a new composite. Different concentrations of cork, TetraPak® and paraffin, different granules size, different temperature and pressure can be used to produce the composite. The electrical properties of the new composite were measured by the isothermal charging and discharging current method. The new composite has shown to have lower conductivity than the commercial agglomerate, which makes it a better material for charge storage [6,13,14,15,16,17,18,19].

### 4.3. Cork/thermoplastic agglomerates

**Figure 4:**
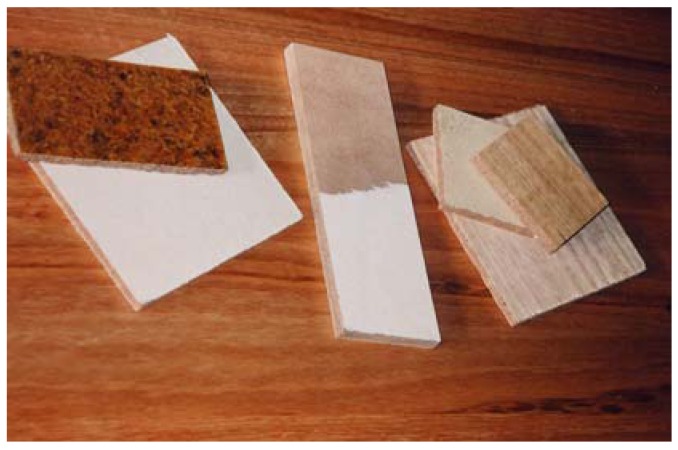
Samples of cork/thermoplastic composites.

An agglomerate of cork particles with thermoplastic binding agents was developed to use mostly cork powder, the major industrial cork waste (see Figure 4). Tests were done using powdery polyethylene (PE) and polypropylene (PP), with *cork powder:thermoplastic* volume ratios of 4:1 and 5:1. Low melting index thermoplastics were chosen due to their advantages over conventional glues, namely the absence of solvents and non toxicity. In the case of some thermoplastics, e.g. polyethylene, the gluing of suitable surface covering sheets is possible in a single agglomeration operation. These new composites are stiff and hard and not resilient, unlike common cork agglomerates, and can be used for panels in multiple applications. The use of other components was also foreseen (e.g. husks and straws) These technologies and products were patented [3,14,15,20,21,22,23,24].

Anther study of PP-cork blends [25] was carried out. A cork surface modification is made to improve cork-matrix adhesion, based on a hot water treatment at room temperature for 1-3 hours and then drying (70 ºC, 3h). Density decreases as function of water treatment time. Tensile tests of the PP matrix reinforced with treated cork shows the importance of this surface modification.

### 4.4. Hydroxypropylcellulose/cork composites

Composites of hydroxypropylcellulose (HPC), a biocompatible polymer, with cork powder, the most important waste in cork processing, offer a new class of materials of interest. Solid films were prepared with different amounts of cork powder (particles < 50 μm) (0.0; 0.5; 1.0 and 10% w/w) and with HPC and a diisocyanate, 1,4-diisocyanatobutane (BDI) (7.0% w/w). When the weight percentage of the cork powder increases the Young’s modulus, the tensile strength and the elongation decrease. However, for the range of concentrations studied, the Young’s modulus for the composite material is higher than for the HPC solid films. For some systems and for the range of concentrations studied, the Young’s modulus for the composite material is smaller than for the HPC crosslinked solid films. The solid films were also characterized by scanning electron microscopy (SEM) and some nucleation points (~0.3 μm) were found [3,26,27,28].

### 4.5. New lignin based resins in cork composite production

The study [29,30] of the agglomeration of cork granules using several external ecological binders based on modified lignin, through operational conditions (pressure, heat and time) sufficient do accomplish a good agglomeration, was carried out. Some of the binders used in composition cork may have toxicological problems (e.g. formaldehyde) and besides this, as the production of cork agglomerates based on cork powder is difficult or even impossible due to its enormous surface area, discovery of an agglomeration technology to overcome these disadvantages would be very important. For the last three decades, the intensive research in the field of lignin ecobinders has been focused on the oxidative enzymes produced by ligninolytic fungi. The production of free radical species as a result of peroxidase and laccase activity is of utmost importance from the industrial point of view: these radicals increase the reactivity of the lignin molecules, leading to further polymerizations in a random non-enzymatic way to form 3-dimensional polymers of higher Mw and with a variety of new linkages. A wide variety of new materials with distinct properties can be thus obtained. The work here presented refers to the development of new ecobinders containing laccase-treated technical lignins, for replacing the synthetic adhesives commonly used in the cork agglomeration industry. For the enzymatic activation of different industrial lignins, different combinations of these lignins and industrial laccase, addition of laccase mediators and organic solvents, and treatment conditions (incubation time, temperature, aeration) were assayed. The ability of these ecobinders to promote granulated cork agglomeration was also determined at lab-scale. Different operational conditions were tested and the agglomerates were physical and mechanically tested. Although some of the first tested binders didn’t work out, a good agglomeration was attained with those containing lignins modified by Novozyme® laccase. Binders having a high viscosity are not suitable to mixture with cork granules (bad distribution). In order to obtain economically/feasible agglomeration conditions, the operational conditions (pressure, pressing time and pressing temperature) should be optimized. As good agglomeration was achieved with short pressing times and low temperature an industrial application is feasible.

### 4.6. Cork powder composite based on the pre-depolymerisation of suberin and polymerization of suberin components

As mentioned above, the production of cork agglomerates based on cork powder is difficult or even impossible due to its enormous surface area. A prior treatment of cork powder for suberin (the major cork chemical component) depolymerisation is carried out using alternating alkaline solutions and acidification followed by a process of elimination of the liquid phase until an adequate degree of dryness is achieved. Afterwards, a hot pressing of this dried material is carried out using several alternative means and operational conditions, for the polymerization of the cork chemical constituents which work like binding agents. Before the pressing stage, the treated cork powder could be mixed with other components, e.g. natural fibers, straws, etc. This process allows the production of cork powder agglomerates which due to its big surface area are difficult or even impossible to produce due to technical and economical problems. Cork powder treatment can be achieved by alkaline hydrolysis in water or in alcohol or by transesterification with low Mw alcohols. The resulting materials are stiff and have a volumetric mass of about 1,000 kg·m^-3^ [24,31].

### 4.7. Synthetic resin/cork material

A Japanese patent application [32] claims a synthetic resin/cork material. This material is formed by stacking an olefinic synthetic resin layer containing cork powder, an olefinic synthetic resin foam body layer and a resin layer in the interior or on the lower surface of the foam body layer. At least, an ethylene-C_4-12_ olefinic copolymer is included in the olefinic synthetic resin layer containing cork powder and in the olefinic synthetic resin foam body layer.

In this field other materials can be mentioned [33]. A foamed polyolefinic resin layer, an adhesive layer and a fibrous layer are successively laminated on a resin layer containing a polyolefinic resin with a bending elastic modulus of 2,000-10,000 kgf/cm^2^, a 15-150 pts·wt per 100 pts·wt of the resin of cork powder with a mean particle size of 200-5,000 μm. The cork powder containing polyolefinic resin laminate can be used as a building member with excellent physical properties, anti-staining properties, cushioning properties, good adhesiveness to a wooden base material and sound insulating properties.

A cork sheet [34] comprising 100 pts·wt of cork granules of 0.6-5 mm and 40-180 pts·wt of an olefinic polymer which comprises an ethylene unit and an alpha-olefin unit greater or equal to C_4_ having a ratio respectively of 55/45 – 99/1 can also be mentioned. This patented material also includes the use of block copolymers and provides a cork sheet which maintains the quality feeling and touch of cork, excellent flexibility and elasticity and good mechanical strength.

### 4.8. Composite corkplate

A composite corkplate is described in a European patent [35]. This material comprises a carrier layer of compacted glues lignocellulosic particles, namely wood chips or fibers and, at least, one covering layer made of glued cork particles. The covering layer is bound to the carrier by simultaneous and mutual compression. This patent also includes a multilayer material which has, at least, one of the exterior surfaces of the plate made of glued cork particles and the middle layer is made of glued lignocellulosic particles. The plate has a density of 0.4-0.8 g/cm^3^.

### 4.9. Cork/charcoal board

A cork/charcoal board was developed for multiple purposes in a Japanese patent [36] which can be related with the improvement of users’ health. A ground cork material is molded into a sheet like shape with a resin binder and powdery or particulate charcoal is incorporated in the cork sheet. This material not only exhibits excellent heat insulating properties, elastic properties, mothproof properties, sound absorbing properties and air permeability, but also effects as a dehumidification action, a deodorizing action, and a negative ion releasing action improving users’ health.

### 4.10. Lightweight polymer mortar with cork granules

Two series of mortar formulations were studied, with different resin/sand (i.e. binder/fine aggregate) weight ratios. In each series cork ranged from 0% to 45% of the total aggregate volume. Flexural and compressive tests were performed. Both the influence of the cork volume fraction and the resin/sand weight ratio were considered relative to the mechanical behavior of the cork modified polymer mortars. A linear decrease in properties was observed as function of the cork volume content. The lower density of the cork modified mortars lead to a smoother loss in specific properties. The results lead to lighter modified polymer concretes with improved compressive ductility [37].

### 4.11. Cork-gypsum composites

Cork and plaster are mutually compatible (there is a good interaction between the gypsum matrix and cork granules) and a lot of new building materials can be made by mixing those materials in different volume fractions. Concerning the acoustical insulation characteristics, this composite is not a sound absorbing material but rather a reflecting one. The thermal insulation properties are quite good. This material is a suggested for use in building applications as partitions. Several types of cork granules can be used and correspond to 10-20% in weight. These composites show a lower density (0.0-1.0 g/cm^3^) when compared with that of similar plasterboard products (>1.2 g/cm^3^). Nevertheless, in order to improve mechanical properties other reinforcing agents are necessary [38].

### 4.12. Polyurethane elastomeric material with cork filler

A study [39] on the effects of fillers, namely cork, on polyurethane resin based polyurethane elastomeric bearing materials for passive isolation was carried out. A series of cork filler incorporated cross-linked molded polyurethane (PU) base on polyethylene adipate diol and 4,4’diphenylmethane diisocyanate with 1,4-butane diol or 1,6-hexanediol and glycerin as chain extender, were synthesized. Mechanical and thermal properties were examined in composites that had between 1 and 15% of cork. The mechanical properties of the composites were found to depend mostly on the amount of filler. The addition of the cork filler into de polyurethane composites yields an increase in Young’s modulus and a decrease in the elongation at break. This new polyurethane-cork composite material with better damping properties can be used as a bearing pad for acoustic and vibration isolation for railway/underground lines.

## 5. Conclusions

A historic survey and the description of current commercial cork composites were carried out. Several cork composites resulting from R&D efforts are now ready to be used. New cork composites can be foreseen, as well as new applications namely due to the specific cork material characteristics. Cork composites are one of the most promising fields of cork technology development.
